# Classification tree analysis for an intersectionality-informed identification of population groups with non-daily vegetable intake

**DOI:** 10.1186/s12889-021-12043-6

**Published:** 2021-11-04

**Authors:** Emily Mena, Gabriele Bolte, on behalf of the Advance Gender study group, Christine Holmberg, Philipp Jaehn, Sibille Merz, Alexander Rommel, Anke-Christine Saß, Kathleen Pöge, Sarah Strasser, Gabriele Bolte, Emily Mena

**Affiliations:** 1 Department of Social Epidemiology, University of Bremen, Institute of Public Health and Nursing Research, Grazer Straße 4, 28359 Bremen, Germany; 2 Health Sciences Bremen, University of Bremen, Bremen, Germany

**Keywords:** Intersectionality, Public health monitoring, Public health reporting, Sex/gender, Gender roles, Vegetable intake, CART, CIT, Health promotion, Public health

## Abstract

**Background:**

Daily vegetable intake is considered an important behavioural health resource associated with improved immune function and lower incidence of non-communicable disease. Analyses of population-based data show that being female and having a high educational status is most strongly associated with increased vegetable intake. In contrast, men and individuals with a low educational status seem to be most affected by non-daily vegetable intake (non-DVI). From an intersectionality perspective, health inequalities are seen as a consequence of an unequal balance of power such as persisting gender inequality. Unravelling intersections of socially driven aspects underlying inequalities might be achieved by not relying exclusively on the male/female binary, but by considering different facets of gender roles as well. This study aims to analyse possible interactions of sex/gender or sex/gender related aspects with a variety of different socio-cultural, socio-demographic and socio-economic variables with regard to non-DVI as the health-related outcome.

**Method:**

Comparative classification tree analyses with classification and regression tree (CART) and conditional inference tree (CIT) as quantitative, non-parametric, exploratory methods for the detection of subgroups with high prevalence of non-DVI were performed. Complete-case analyses (*n* = 19,512) were based on cross-sectional data from a National Health Telephone Interview Survey conducted in Germany.

**Results:**

The CART-algorithm constructed overall smaller trees when compared to CIT, but the subgroups detected by CART were also detected by CIT. The most strongly differentiating factor for non-DVI, when not considering any further sex/gender related aspects, was the male/female binary with a non-DVI prevalence of 61.7% in men and 42.7% in women. However, the inclusion of further sex/gender related aspects revealed a more heterogenous distribution of non-DVI across the sample, bringing gendered differences in main earner status and being a blue-collar worker to the foreground. In blue-collar workers who do not live with a partner on whom they can rely on financially, the non-DVI prevalence was 69.6% in men and 57.4% in women respectively.

**Conclusions:**

Public health monitoring and reporting with an intersectionality-informed and gender-equitable perspective might benefit from an integration of further sex/gender related aspects into quantitative analyses in order to detect population subgroups most affected by non-DVI.

**Supplementary Information:**

The online version contains supplementary material available at 10.1186/s12889-021-12043-6.

## Background

Frequent and high intake of vegetables is considered an important behavioural health resource, associated with improved immune function [1] and lower incidence of non-communicable disease [2]. Empirical evidence from an umbrella review, including results based on cohort studies, supports particular protective effects in light of all-cause mortality, cardiovascular diseases, cancer and depression [3]. In line with this, insufficient vegetable intake has been shown to be associated with specific diseases such as haemorrhagic and ischaemic stroke, oesophageal cancer and ischaemic heart disease [4].

Using population-based data sources, Stea et al. [5] examined vegetable consumption according to gender, educational attainment and regional affiliation in Europe and found that being female and having a high educational status was associated with increased vegetable intake. Focusing on sex/gender as the most prominent characteristic discussed for non-daily vegetable intake at the population-scale [5–7], differences between women and men seem to remain quite stable across age and educational groups [6, 7] as well as marital status and place of residence [6].

Public health promotion with the aim to increasing vegetable and fruit consumption is based on recommendations for at least 400 g of edible vegetables and fruit per day, and is part of a global strategy with targeted campaigns and programs being strongly encouraged by the WHO [8]. The National Cancer Institute (NCI), the national health authority and lead federal agency for the campaign in the U.S., has initiated key initiatives to increase consumption of fruit and vegetables mainly by targeting men and increasing availability of fruits and vegetables in schools. Based on the observation of higher prevalence of non-daily vegetable intake in men when compared to women, men are prioritized when defining certain population groups as proper recipients for health promoting interventions [7].

However, relying on the male/female binary in quantitative analyses as in the aforementioned studies might conceal heterogeneity within the categories male or female [9]. Taking such intragroup differences into account might be crucial for the development of intervention strategies. This has been illustrated by the work of legal scholar Kimberle Crenshaw. Crenshaw (1989) coined the term intersectionality to describe the ways in which experiences of discrimination interact for individuals who are positioned at the intersection of different social systems or identities. In the context of identity politics and social justice, she developed an argument focusing on the intersections of gender, race and class domination, centring the experiences of women of colour living in the U.S.. Crenshaw (1989) elaborated on how intervention strategies which are based exclusively on experiences of women are of limited help to women of colour who, because of the intersections of gender, race and class, face different obstacles than white women. Since women of colour in the U.S. are often confronted with poverty, childcare responsibilities and the lack of formal vocational training, the lived experiences of this population group were likely to be different when compared to other female population groups (Crenshaw 1989). Meanwhile, the concept of intersectionality is gaining popularity across different disciplines (Bauer 2021) including public health (Bowleg 2012). Most notably, Hancock (2007), McCall (2005) and Bauer (2021) have advanced the translation of the principles of intersectionality into quantitative research. Taken together, it can be emphasised that incorporating the concept of intersectionality into quantitative health analyses might help uncovering the distribution of the burden of disease across social locations by taking intersections of socially driven differences into account [10]. Consequently, the consideration of socio-cultural, socio-demographic and socio-economic factors in conjunction with further sex/gender related aspects in multivariable analyses based on data drawn from population-based studies could assist in unmasking some of the possible sex/gender related heterogeneity in non-daily vegetable intake. This heterogeneity might be overlooked when relying merely on the binary sex/gender variable. Decentralising a trait like “being” male or female and considering other sex/gender related aspects such as gender roles, which are transformable social factors, might strengthen public health monitoring and reporting and its sex/gender sensitivity. As a consequence, a more differentiated understanding of heterogeneity between and within the groups of men and women might be attained. This could serve as a fertile ground for the development and implementation of targeted interventions for the promotion of daily vegetable intake, incorporating a gender equitable perspective.

From a statistical point of view, classification tree analysis is a quantitative, non-parametric, exploratory method suitable for analysis of interactions from an intersectionality-informed perspective, which can support the detection of subgroups with higher or lower prevalence of certain diseases or related risk factors [11, 12]. In contrast to other parametric procedures, classification tree analysis makes no distributional assumptions, neither on the outcome nor on the predictor variables, and is not affected by collinearities, outliers, heteroscedasticity, or distributional error structures [13]. There are various decision tree algorithms available, for example the commonly used “Classification and Regression Tree” (CART) [11], and the “Conditional Inference Tree” (CIT) [14]. Even though classification tree methods were originally developed to be able to deal with low sample sizes when analysing rare diseases in the medical field [11, 15], they are increasingly being recommended for analysis of data collected for surveillance purposes as well [16]. Therefore, it is not surprising that the application of classification trees is increasing in public health [16]. While comparisons of prevalence rates are most commonly analysed across strata of only one or two independent variables, especially in public health monitoring and reporting, classification trees allow for making better use of available surveillance data by facilitating the analysis of a variety of independent variables simultaneously [16]. Accordingly, use of classification trees may support a more precise identification of population groups that are heterogeneous in terms of non-daily vegetable intake. So far, however, classification tree analysis of population-based data to identify population groups differing in prevalence of non-daily vegetable intake seem to be scarce and, if conducted, are done without comprehensively considering sex/gender related aspects such as gender roles [17, 18]. Consequently, the aim of our present analysis was (1) to explore possible interactions of sex/gender or sex/gender related aspects with a variety of different socio-cultural, socio-demographic and socio-economic variables from an intersectionality-informed perspective and (2) to compare the results of two different decision tree algorithms. In the present study, we based our analyses on comprehensive public health monitoring data from Germany and compared the results of CART and CIT for building classification trees with non-daily vegetable intake as the outcome.

## Methods

### Study sample

The present study is based on the National Health Telephone Interview Survey ‘GEDA - German Health Update’ [GEDA 2009]. This cross-sectional, representative survey is repeatedly conducted by the Robert Koch Institute (RKI) as part of a nationwide public health monitoring system for diseases and health behaviour. The random sample of telephone numbers for the computer-assisted telephone interviews of the GEDA survey were drawn according to the Gabler-Häder method [19]. The participants of GEDA 2009 were randomly selected, German speaking adults (age range: 18–100 years), who were registered in Germany (*n* = 21,262). The cooperation rate for participants, measured as the proportion of realised interviews with individuals that have been contacted was 51.2% [19]. Further details about design, methods and nationwide representativeness of the study population of the GEDA 2009 survey have been described elsewhere [20]. The final study sample of the present study comprised 19,512 participants overall.

### Health-related outcome: non-daily vegetable intake (non-DVI)

Frequency of vegetable intake was assessed by asking study participants the following question: How often do you eat vegetables? The following answer categories were offered: every day, at least once a week, less than once a week, never/ I do not know. The variable for vegetable intake in the present study was dichotomized into daily (every day) vs non-daily (at least once a week, less than once a week, never/ I do not know).

### Socio-cultural, socio-demographic and socio-economic variables

We selected the following nine variables of GEDA 2009, capturing different sociocultural, socio-demographic and socio-economic dimensions: Sex/gender [forced choice: female, male], Age [in years: 18–29, 30–39, 40–49, 50–59, 60–69, 70–79, 89+]; Education [categorized according to ISCED 1997 EU-classification: high, medium, low]; Employment status [full-time, part-time, occasionally, not working], Professional status [blue-collar worker, white-collar worker, civil servant, freelancer, helping family, no profession, else]; Marital status [married, married - living separately, unmarried, divorced, widowed]; Disability status [yes, no]; Migration background [21] [two-sided (non-German citizenship, respondent immigrated to Germany after birth or both parents not born in Germany), one-sided (one parent not born in Germany), no (without migration background)]; Urbanity/rurality [big city, city, rural, very rural].

### Sex/gender related aspects

In reference to a gender concept which has been developed at the Canadian Institutes of Health Research and has also served as a basis for the development of a composite measure of gender [22, 23], we defined six available variables of GEDA 2009 as sex/gender related aspects indicating possible mechanisms underlying sex/gender differences in health [24, 25]: Family constellation [with partner and child(ren), with partner and no child(ren), no partner and with child(ren); no partner and no child(ren)]; Main earner status [main wage earner in the household: one person household, respondent herself/himself, partner, other, there is no main wage earner]; Perceived social support measured by the 3-item Oslo Scale (low, medium, high) [26, 27]; Burden due to household responsibilities [5-point Likert-scale: 1 (not at all), 2, 3, 4, 5 (a lot)]; Burden due to childrearing responsibilities [5-point Likert-scale: 1 (not at all), 2, 3, 4, 5 (a lot)]; Burden due to informal care responsibilities [5-point Likert-scale: 1 (not at all), 2, 3, 4, 5 (a lot)].

### Statistical analysis

The complete case analyses were based on data from study participants of GEDA 2009 providing information about the frequency of vegetable intake and relevant covariables (total sample *n* = 19,512, 8381 men and 11,156 women). We pursued two analytical strategies with different combinations of the binary sex/gender variable, socio-cultural, socio-demographic and socio-economic variables as well as sex/gender related aspects. In strategy 1 we included socio-cultural, socio-demographic and socio-economic variables and the binary sex/gender variable. In strategy 2 we included socio-cultural, socio-demographic and socio-economic and sex/gender related aspects and subsequently calculated proportions of men and women within the identified subgroups of the model. First, descriptive statistics of all covariables and the prevalence of non-DVI within each category were computed. Second, in order to detect subgroups with differences in prevalence of non-DVI, the classification task was performed with CART [11] and CIT [14] as decision tree building algorithms using the rpart package [28], partykit package [14, 29] and R 3.6.1 [30]. The CART algorithm represents a popular decision tree technique, which has been criticised for not having a concept of statistical significance. The newer CIT-approach follows formal statistical procedures in each splitting step [14, 31]. Since no straightforward recommendation is available on which algorithm and specifications for complexity parameter (cp) in CART or α-level in CIT should be preferred in light of public health monitoring data, we compared different specifications in our analyses by calculating in total 8 classification trees, 4 for each of the aforementioned strategies. The 2 CART-analyses were conducted using either cp = 0.01 (default specification in R) or cp = 0.005. The selection of the final tree model was based on the cross-validation estimates of the error of the sub-trees of the initial tree, along with the standard errors of the respective estimates. Correspondingly, fitted trees were ‘pruned’ according to the 1-SE rule to develop a tree with the best size and lowest misclassification rate by selecting the least complex tree whose error was within one standard error above the tree with the smallest cross-validated error [11]. The 2 CIT-analyses were conducted using either α-level = 1% or α-level = 0.1% both with Bonferroni correction. In order to keep the size of the tree and the results interpretable, we did not use the default specification in R of α-level = 5%. In all models, cost weights were assigned to equally distribute sums of weights for cases and non-cases, thereby assigning equal importance to sensitivity and specificity [32]. The minimum node size allowed in a terminal node was restricted to contain at least 1% of the overall analysis population. No other survey-specific weighting factors were applied. Unweighted percentage of population as well as prevalence of non-DVI were calculated for each node separately. In strategy 2, which does not include the sex/gender binary as a covariable, we calculated proportions of men and women within the identified subgroups of the model. Subsequently we computed prevalence of non-DVI for men and women separately as well as the resulting absolute difference in prevalence.

## Results

The study population (*n* = 19,512) comprised overall 50.9% individuals with non-DVI (at least once a week: 46.9%, less than once a week: 3.5%, never/ I do not know: 0.4%). Prevalence in the male population (*n* = 8372) was 61.7%, prevalence in the female population (*n* = 11,140) 42.7%, respectively. Socio-cultural, socio-demographic and socio-economic of the study population and the prevalence of non-DVI within categories of these characteristics are shown in Table 1. Prevalence of non-DVI ranged overall between 40.6–62.4%. Lowest prevalence of non-DVI across categories of all socio-cultural, socio-demographic and socio-economic (range: 40.6 - 48.9%) was found for helping family members, women, working part-time, freelancer, high education, individuals aged 60+, having a two-sided migration background, being married or living in a big city. Comparison of individuals with or without disability status showed almost no difference in prevalence with approximately 51% for both subgroups. Highest prevalence across categories of all socio-cultural, socio-demographic and socio-economic variables (range: 62.4 - 51.3%) was found for blue-collar workers, men, low education, being unmarried or divorced, living in a very rural area, individuals under 60 years of age or having no migration-background. A gradient with monotonously increasing prevalence of non-DVI across categories was visible with regard to education or migration background.

**Table 1:** Socio-cultural, socio-demographic and socio-economic characteristics of the study population and prevalence of non-daily vegetable

Socio-cultural, socio-demographic and socio-economic variables	Proportion of characteristic % (n)	Prevalence of non-daily vegetable intake % (n)
N	100 (19512)	
Sex/gender		
Female	57.09 (11140)	42.67 (4753)
Male	42.91 (8372)	61.73 (5168)
Age		
18–29	17.72 (3458)	53.82 (1861)
30–39	16.22 (3164)	49.46 (1565)
40–49	23.40 (4565)	51.59 (2355)
50–59	17.53 (3420)	52.02 (1779)
60–69	14.32 (2795)	47.69 (1333)
70–79	8.29 (1618)	48.52 (785)
80+	2.52 (492)	49.39 (243)
Education		
Low	9.56 (1865)	56.57 (1055)
Middle	51.37 (10023)	53.98 (5410)
High	39.07 (7624)	45.33 (3456)
Employment status		
Not working	36.17 (7058)	48.21 (3403)
Full-time	42.40 (8274)	56.71 (4692)
Part-time	16.91 (3300)	43.09 (1422)
Occasionally	4.51 (880)	45.91 (404)
Professional status		
No profession	1.80 (1532)	54.77 (839)
Blue-collar	15.84 (3090)	62.43 (1929)
White-collar	55.99 (10925)	49.07 (5361)
Official	7.51 (1465)	46.48 (681)
Freelancer	10.00 (1951)	44.18 (862)
Helping family	1.01 (197)	40.61 (80)
Else	7.85 (352)	48.01 (169)
Marital status		
Married	53.58 (10455)	48.05 (5024)
Married - living separately	2.60 (508)	53.94 (274)
Unmarried	27.45 (5357)	54.88 (2940)
Divorced	8.82 (1721)	54.45 (937)
Widowed	7.54 (1471)	50.71 (746)
Disability status		
Yes	8.12 (1585)	51.36 (814)
No	91.88 (17927)	50.80 (9107)
Migration background		
No	85.62 (16706)	51.30 (8571)
One-sided	3.88 (757)	49.67 (376)
Two-sided	10.50 (2049)	47.54 (974)
Urbanity/rurality		
Big city	31.06 (6060)	48.89 (2963)
City	39.43 (7694)	51.38 (3953)
Rural	15.46 (3016)	51.33 (1548)
Very rural	14.05 (2742)	53.14 (1457)

**Fig. 1:**
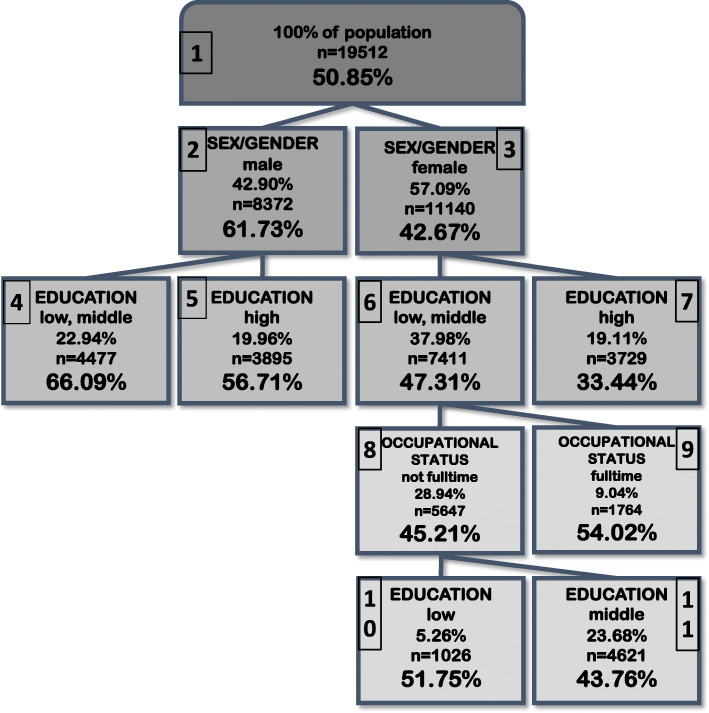
Strategy 1 - Splitting variables, proportion of study population and prevalence of non-DVI within subgroups detected by CIT-analysis (α-level = 1%) based on binary sex/gender variable and socio-cultural, socio-demographic and socio-economic variables of the full sample

Figure 1 shows the splitting variables, the proportion of the study population and the prevalence of non-DVI within subgroups (nodes) detected by CIT-analysis with α-level = 1% including binary sex/gender, socio-cultural, socio-demographic and socio-economic variables. The first split in strategy 1 was induced by sex/gender. The prevalence of non-DVI ranged overall between 33.4 -66.1% in the identified subgroups. Prevalence in men ranged between 56.7 -66.1%. Prevalence in women ranged between 33.4 -54.1%. Prevalence of non-DVI was higher in men across all nodes of the tree when compared to women. In women, the highest prevalence of non-DVI of 54.1% occurred in women with low or middle education who are employed full-time. The lowest prevalence of 33.4% was found for women with high education. In men, the highest prevalence of non-DVI of 66.1% occurred in men with low or middle education. The lowest prevalence of 56.7% was found for men with high education. The CIT with α-level = 0.1% (see Additional file 1) gave the same results as shown in Fig.1 except for the last split for education in women (nodes 10,11), which was only observed in the CIT analysis with α-level = 1%. The trees based on the CART-algorithm with cp = 0.01 and cp = 0.005 (see Additional file 2) showed only the first split by sex/gender (nodes 2,3).

Table 2 shows prevalence of non-DVI within subgroups characterized by sex/gender related aspects. Prevalence of non-DVI ranged overall between 40.5 - 56.5%. Lowest prevalence across categories of all sex/gender related aspects ranged between 40.5–46.6%: A prevalence of approximately 41% occurred in individuals who share a household with a partner who is the main earner or for individuals who feel strongly burdened by childrearing responsibilities, respectively. A prevalence of approximately 46% was found for individuals with no partner but with child(ren), for individuals who feel rather burdened by household or moderately burdened by care activities or for individuals with perceived high social support, respectively. Highest prevalence across categories of all sex/gender related aspects ranged between 56.5 - 52.2%: A prevalence of approximately 56% was found for individuals with perceived low social support, for individuals living alone, sharing a household with the partner and being the main earner of the household (independent of other individuals living in the household) or for individuals with no partner and no child(ren), respectively. A prevalence of approximately 53% was found for individuals with no children (independent of the partner status) or for individuals who did not feel burdened by household or care responsibilities, respectively. The lowest range in prevalence within sex/gender related aspects appeared for care or household burden. The highest range in prevalence within sex/gender related aspects was found for main earner status. A gradient with monotonously increasing prevalence of non-DVI across categories was visible with regard to childrearing burden or social support.

**Table 2:** Sex/gender related characteristics of the study population and prevalence of non-daily vegetable intake

Socio-cultural, socio-demographic and socio-economic variables	Proportion of characteristic % (n)	Prevalence of non-daily vegetable intake % (N)
N	100 (19512)	
Family constellation		
No partner, no child(ren)	33.85 (6604)	55.03 (3634)
Partner, no child(ren)	37.41 (7300)	49.56 (3618)
Partner, child(ren)	24.88 (4854)	47.75 (2318)
No partner, child(ren)	3.86 (754)	46.55 (351)
Main earner		
1 Person household	21.94 (4281)	55.64 (2382)
Respondent	31.10 (6068)	55.83 (3388)
Partner	26.78 (5225)	40.50 (2116)
Another person	8.87 (1730)	54.05 (935)
None	11.32 (2208)	49.82 (1100)
Household burden		
Not applicable	1.09 (212)	50.47 (107)
1 (not at all)	26.69 (5208)	52.25 (2721)
2	28.91 (5640)	51.86 (2925)
3	28.02 (5468)	50.24 (2747)
4	8.64 (1686)	46.33 (803)
5 (a lot)	6.65 (1298)	47.61 (618)
Childrearing burden		
No child(ren)	26.36 (5144)	53.34 (2744)
1 (not at all)	38.92 (7595)	51.78 (3933)
2	13.55 (2643)	49.83 (1317)
3	11.56 (2256)	49.29 (1112)
4	11.32 (997)	45.14 (450)
5 (a lot)	4.49 (877)	41.62 (365)
Care burden		
No care tasks	48.59 (9480)	50.78 (4814)
1 (not at all)	35.55 (6937)	52.43 (3637)
2	5.90 (1151)	47.52 (547)
3	4.61 (900)	46.33 (417)
4	2.62 (511)	48.14 (246)
5 (a lot)	2.73 (533)	48.78 (260)
Social support		
High	34.26 (6684)	46.51 (3109)
Middle	51.42 (10034)	52.15 (5233)
Low	14.32 (2794)	56.51 (1579)

**Fig. 2:**
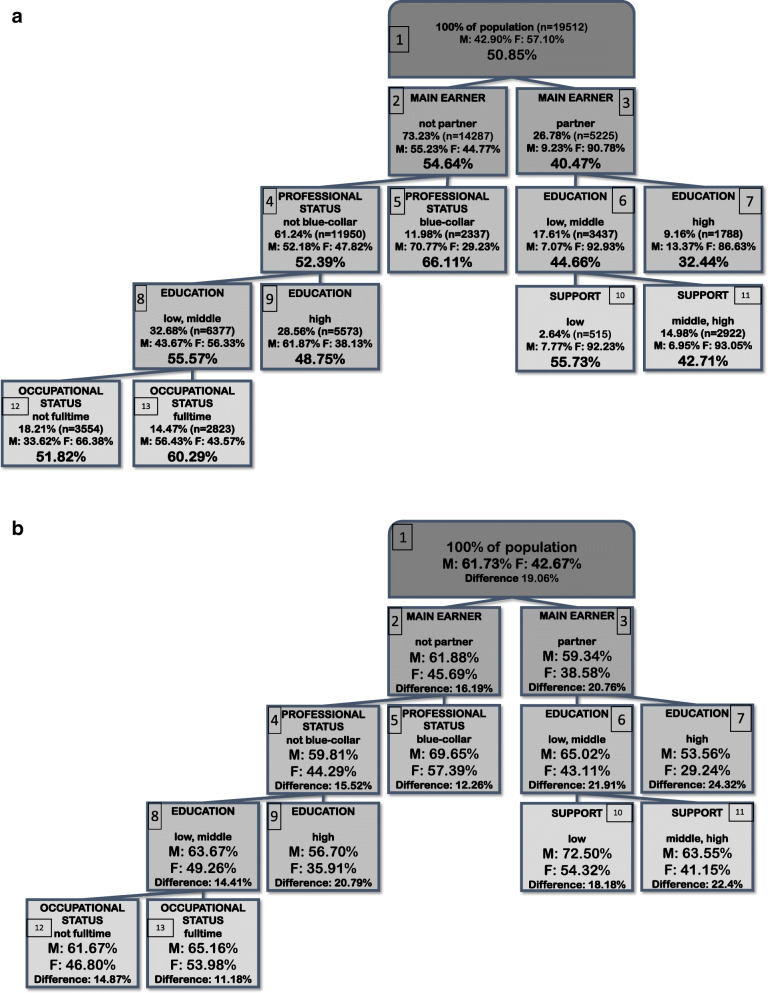
**a** Strategy 2 - Splitting variables, proportion of study population and prevalence of non-DVI within subgroups detected by CIT-analysis (α-level = 1%) based on sex/gender related aspects and socio-cultural, socio-demographic and socio-economic variables of the full sample. **b** Splitting variables of the CIT-analysis (α-level = 1%; Fig. 2a) based on sex/gender related aspects and socio-cultural, socio-demographic and socio-economic variables of the full sample (Fig. 2a) with prevalence of non-DVI stratified by male/female

Figure 2a shows the splitting variables, the proportion of study population and the prevalence of non-DVI within subgroups (nodes) detected by CIT-analysis with α-level = 1% (strategy 2). Proportion of men and women within the identified subgroups were added as additional information to the classification tree. The prevalence of non-DVI ranged overall between 32.4 -66.1%. The first split in strategy 2 was induced by main earner status, dividing the sample into a branch with individuals who share a household with a partner who is the main earner (26.8% of the total sample) and a branch with individuals who do not share a household with a partner who is the main earner [73.3% of the total sample: individuals living alone, sharing the household at least with a partner but being the main earner themselves, sharing the household at least with one other person but with no one being a main earner and sharing the household at least with one other person who is the main earner]. For individuals who live with a partner and whose partner is the main earner in the household, the highest prevalence of non-DVI of 55.7% was found in individuals who have low or middle education and who perceive their social support to be low. The same prevalence was found for individuals who do not share a household with a partner who is the main earner, who have low or middle education and are not blue-collar workers. The lowest prevalence for individuals whose partner is the main earner in the household of 32.4% occurred in individuals with high education. For individuals who do not share a household with a partner who is the main earner, the highest prevalence of non-DVI was 66.1% and was found for blue-collar workers. Second highest prevalence of non-DVI of 60.3% occurred in individuals who do not work as a blue-collar but have low or middle education and are employed full-time. The trees using the CART-algorithm with either cp = 0.01 or cp = 0.005 (see Additional file 3) were restricted to the split by main earner status, followed by further splits only for individuals whose partner is not the main earner in the household by professional and then educational status (nodes 2,3,4,5,8,9). The CIT with α-level = 0.1% showed the same results as shown in Fig. 2a.

In Fig. 2b the information about prevalence of non-DVI within the identified subgroups of the classification tree analysis shown in Fig. 2a is extended by an additional calculation of prevalence of non-DVI separately for men and women and by indication of absolute difference in prevalence between males and females. The prevalence of non-DVI ranged overall between 29.2 - 72.5%, in men between 53.6 - 72.5%, and in women between 29.2 - 57.4%. The highest prevalence of 72.5% occurred in men whose partner is the main-earner in the household, who have low or middle education and who perceive their social support to be low. This node represents 2.6% of the total study sample and is by far the smallest detected population subgroup with 515 individuals of which only 7.8% are men. Second highest prevalence in men and highest prevalence in women was found for individuals, who do not share a household with a partner who is the main earner and who work as a blue-collar (men: 69.7%, women: 57.4%; absolute difference in prevalence: 12.3%). This node represents 12% of the total study sample of which only one third are women. In both cases the prevalence within men as well as within women is higher than the highest prevalence detected in strategy 1. Lowest prevalence in men and women was found for individuals whose partner is the main-earner in the household and who have high education (men: 53.6%, women: 29.2%, absolute difference in prevalence: 24.3%). In both cases the prevalence within men and within women is lower than the lowest prevalence detected in strategy 1. In general, the proportion of men in all nodes of the branch with individuals who live with a partner in the household and whose partner is the main earner does not exceed 13.4% (with at least 40 but not more than 482 men within a node). The subgroups of the branch with individuals whose partner is the main earner in the household show for the most part higher differences in prevalence (nodes 3, 6, 7, 11) than the difference between men and women in the root node (19.1%). The subgroups of the branch with individuals who do not share a household with a partner who is the main earner predominantly show lower differences in prevalence (nodes 2, 4, 5, 8, 12, 13) than the difference between men and women in the root node (19.1%).

## Discussion

Sex/gender differences in vegetable intake are widely documented, but not well understood [33]. In the present study we applied classification tree analysis in order to unmask heterogeneity behind the male/female binary by exploring how interactions of different socio-cultural, socio-demographic and socio-economic variables in light of non-DVI might differ when either considering the binary sex/gender variable or various sex/gender related aspects instead. In strategy 1 subgroups with the highest or lowest prevalence were mainly characterised by interactions between the sex/gender binary and education. In this regard, the results of our explorative intersectional approach were in line with previous findings, which showed that mainly men and individuals with low education have the lowest frequency in vegetable intake [5, 7]. In turn, in strategy 2 considering sex/gender relevant variables instead of the sex/gender binary variable, subgroups with highest prevalence were characterised by interactions between main earner status and professional status and lowest prevalence by main earner status and education respectively.

Diverging from other analyses with population-based data which have shown that differences in non-DVI between men and women remained stable across age groups [6, 7], the results of the classification tree analyses suggest that variables related to socio-economic status might be more relevant than age for the characterisation of subgroups with higher prevalence of non-DVI, when viewed from an intersectional perspective. Furthermore, different indicators of socio-economic status seem to interact more strongly in women when compared to men: In strategy 1, women with low or middle education who work full-time showed highest prevalence of non-DVI, whereas in men educational status did not interact with occupational status. In strategy 2, the subgroups resulting from splits based on the variables associated with socio-economic status such as education, professional status and occupational status showed larger differences in prevalence within the group of women when compared to men. The results suggest that socio-economic status represents an important category of difference when aiming at the detection of heterogeneity, primarily in women. In dietary research including investigations about vegetable intake, the use of separate indicators of socio-economic status such as education and occupation has been strongly recommended since they seem to reflect different underlying social processes [34, 35]. With this in mind, it is likely that including a variable such as main earner status into the classification tree analysis might have partly uncovered the more complex nature of the interrelatedness of different indicators of socio-economic status with regard to non-DVI, especially in women.

In view of the comparison of the 2 different classification tree algorithms, the trees constructed by CART were, overall, noticeably smaller compared to the trees constructed by CIT. All the subgroups detected by CART were also detected by CIT. Comparing the results of both strategies applied in the present study, most heterogeneity became visible especially within the group of women: In line with Stea et al. [5], in strategy 1 women with high education were identified as the subgroup with the lowest prevalence of non-DVI. In contrast, by considering sex/gender relevant aspects in strategy 2, only women with high education who live with a main earner as a partner were detected with a prevalence of 29.2%. This is by far the lowest prevalence when compared to the total study population. Consequently, more than 10% of the entire study population, which were represented as the subgroup women with high education in strategy 1 (Fig. 1, node 7), has been distributed across other subgroups in strategy 2 with consistently higher prevalence of non-DVI than identified within the group of women with high education. Finally, the results of strategy 2 clarified that especially blue-collar men and women who do not share a household with a partner who is the main earner are the most affected population group with regard to non-DVI. Not least, this finding seems plausible because higher levels of stress, which have shown to be more pronounced in blue-collar workers when compared to white collar workers, were found in previous research to be associated with lower vegetable intake [36–38].

To our knowledge, when analysing sex/gender differences in non-DVI in population-based studies, the role of main earner status has not yet been emphasized explicitly. However, prevailing sex/gender differences in shared responsibilities with regard to labour participation and homemaking are widely recognized [39, 40] and are presumed to constitute different epidemiological patterns of privilege and disadvantage with regard to health of men and women [41]. The low proportion of men who share a household with a partner who is the main earner might point to main earner responsibilities being more strongly embodied in male identity, while the opposite status could be seen as being more embodied in female identity, at least to a certain extent: Roundabout 25% of the total study population are women who have a partner in the household on whom they can rely on financially, of which one third are women with high education and show by far the lowest prevalence in non-DVI. Not differentiating by main earner status might be responsible for the masking of heterogeneity within the group of women with regard to non-DVI, due to focusing merely on the binary sex/gender variable in multivariable analysis. Consequently, the group of women most affected by high prevalence of non-DVI, which represents 3.5% of the total study population, might be overlooked as an important target group for the implementation of health promoting interventions: Compared to women with high education living with a partner in the household who has main earner responsibilities, the prevalence of non-DVI in blue-collar women who do not share a household with a partner who is the main earner is twice as high. Furthermore, the non-DVI prevalence of 57.4% in these blue-collar women is quite comparable to the prevalence in the entire group of men as shown in strategy 1.

Especially in view of research on health of blue-collar women, results from a systematic review evaluating respective research over the past quarter century suggest that blue-collar women in general experience worse health when compared to blue-collar men or women with other professional status [42]. Furthermore, it seems plausible that main earner status, working full-time or working as a blue-collar could be linked to higher levels of stress [38, 43, 44] and at the same time represent drivers of difference in prevalence of non-DVI across categories of sex/gender. These factors are likely to reflect aspects of gender roles more strongly, but not exclusively, internalized by men when compared to women.

Finally, the results of strategy 2 suggest that those blue-collar men and women, who do not share a household with a partner who is the main earner, are most affected by non-DVI. This subgroup represents overall 12% of the total study population. Even though women in this specific subgroup represent overall no more than 3.5% of the study population, they show the highest prevalence by far of non-DVI when compared to other women of the total sample. By not additionally considering sex/gender relevant aspects beyond the male/female binary, these women might have been overlooked as possibly suitable recipients for health promoting targeted interventions.

### Strength and limitations

There are some limitations to consider, when interpreting the results of the present study. Respondents in health surveys show more positive health-related behaviours compared to non-respondents [45] and therefore the prevalence of non-DVI might have been underestimated. As there were only low numbers of individuals never consuming vegetables or less than once a week, we only compared daily vegetable intake to less than daily intake. Due to data availability, we were not able to include information about quantity of vegetable intake. Furthermore, it might be criticized that the data of GEDA 2009 used in this study are quite outdated. However, given that the aim of this study was to test a theory-based methodological approach, the GEDA 2009 survey provided a higher number of variables that describe sex/gender relevant mechanisms such as gender roles compared to other more recent surveys of the RKI in Germany. Overall, men showed higher prevalence of non-DVI than women within all identified subgroups of both strategies, which points to the binary sex/gender variable as the most strongly classifying variable. It was not possible to include further sex/gender related aspects than the ones studied, which might have unmasked more of the assumed heterogeneity in prevalence within and between the group of men and women. To name a few, consideration of factors such as drive for thinness [46], favourable attitudes, perceived behavioural control over frequent vegetable intake [33], being the primary meal planner or feeling responsible for the family’s overall health [47] might have further lowered the remaining difference in prevalence of non-DVI. Furthermore, by not including the binary sex/gender as an analytical category into the multivariable analysis in strategy 2, we were able to move beyond a traditional binary male/female approach only when viewed from a methodological perspective. Unfortunately, due to data availability, we did not have the opportunity to explore other non-binary concepts of gender identity, which embrace further identities such as trans or gender queer identities. Nevertheless, strategy 2 opens up the possibility to push public health monitoring and reporting past a traditional binary sex/gender approach to gender identity, since the strategy allows the description of proportions and prevalence within the identified subgroups by several gender groups if data on gender identities are available.

A strength of our analysis is the use of classification trees as an exploratory method to identify subgroups, which substantially differ in prevalence of non-DVI [16]. To this end, we compared the results of different algorithms and specifications of CART and CIT. We found that the main findings were quite robust, since the same subgroups were identified by both algorithms and all specifications alike. Finally, another strength of our study is the inclusion of a wide range of socio-cultural, socio-demographic and socio-economic variables as well as sex/gender relevant aspects based on data of a national survey on health of adults in Germany.

## Conclusion

The inclusion of sex/gender related aspects in multivariable analysis as part of an intersectionality-informed and sex/gender sensitive strategy supported the unmasking of heterogeneity within and between the groups of men and women. The identification of subgroups with higher prevalence of non-DVI, which might be overlooked when focusing exclusively on the male/female binary instead of other sex/gender relevant aspects, could contribute to the tailoring of targeted interventions from a more gender-equitable perspective: Especially blue-collar women who do not share a household with a partner who is the main earner showed highest prevalence compared to other women and lowest difference in prevalence when compared to men. Not including other sex/gender related aspects than the binary sex/gender variable might have concealed this heterogeneity primarily within the group of women. In conclusion, taking additional sex/gender related aspects in light of non-DVI into account, especially in public health monitoring analyses, could result in further lowering the preference of the binary sex/gender variable as a strongly differentiating factor. The variable, when used in isolation, carries the risk of concealing existing heterogeneity within and between the groups of men and women. While the consideration of sex/gender as a binary individual characteristic has already been successfully implemented as a standard approach in public health reporting [48], an integration of more sex/gender related aspects in explorative data analyses could be the next step to further improve sex/gender sensitivity in public health monitoring and reporting.

## Supplementary Information

**Additional file 1.**

**Additional file 2.**

**Additional file 3.**

## Data Availability

The dataset of the GEDA 2009 study [19] are available as public use file for scientific use from fdz@rki.de. Further information: https://www.rki.de/DE/Content/Gesundheitsmonitoring/Studien/Geda/Geda_2009_inhinh.html;https://www.rki.de/DE/Content/Forsch/FDZ/Zugang/SUF.html

## References

[1] Hosseini B, Berthon BS, Saedisomeolia A, Starkey MR, Collison A, Wark PAB, Wood LG (2018). Effects of fruit and vegetable consumption on inflammatory biomarkers and immune cell populations: a systematic literature review and meta-analysis. Am J Clin Nutr.

[2] Kalmpourtzidou A, Eilander A, Talsma EF (2020). Global vegetable intake and supply compared to recommendations: a systematic review. Nutrients..

[3] Angelino D, Godos J, Ghelfi F, Tieri M, Titta L, Lafranconi A, Marventano S, Alonzo E, Gambera A, Sciacca S, Buscemi S, Ray S, Galvano F, del Rio D, Grosso G (2019). Fruit and vegetable consumption and health outcomes: an umbrella review of observational studies. Int J Food Sci Nutr.

[4] Forouzanfar MH, Afshin A, Alexander LT, Anderson HR, Bhutta ZA, Biryukov S, Brauer M, Burnett R, Cercy K, Charlson FJ, Cohen AJ, Dandona L, Estep K, Ferrari AJ, Frostad JJ, Fullman N, Gething PW, Godwin WW, Griswold M, Hay SI, Kinfu Y, Kyu HH, Larson HJ, Liang X, Lim SS, Liu PY, Lopez AD, Lozano R, Marczak L, Mensah GA, Mokdad AH, Moradi-Lakeh M, Naghavi M, Neal B, Reitsma MB, Roth GA, Salomon JA, Sur PJ, Vos T, Wagner JA, Wang H, Zhao Y, Zhou M, Aasvang GM, Abajobir AA, Abate KH, Abbafati C, Abbas KM, Abd-Allah F, Abdulle AM, Abera SF, Abraham B, Abu-Raddad LJ, Abyu GY, Adebiyi AO, Adedeji IA, Ademi Z, Adou AK, Adsuar JC, Agardh EE, Agarwal A, Agrawal A, Kiadaliri AA, Ajala ON, Akinyemiju TF, al-Aly Z, Alam K, Alam NKM, Aldhahri SF, Aldridge RW, Alemu ZA, Ali R, Alkerwi A', Alla F, Allebeck P, Alsharif U, Altirkawi KA, Martin EA, Alvis-Guzman N, Amare AT, Amberbir A, Amegah AK, Amini H, Ammar W, Amrock SM, Andersen HH, Anderson BO, Antonio CAT, Anwari P, Ärnlöv J, Artaman A, Asayesh H, Asghar RJ, Assadi R, Atique S, Avokpaho EFGA, Awasthi A, Quintanilla BPA, Azzopardi P, Bacha U, Badawi A, Bahit MC, Balakrishnan K, Barac A, Barber RM, Barker-Collo SL, Bärnighausen T, Barquera S, Barregard L, Barrero LH, Basu S, Batis C, Bazargan-Hejazi S, Beardsley J, Bedi N, Beghi E, Bell B, Bell ML, Bello AK, Bennett DA, Bensenor IM, Berhane A, Bernabé E, Betsu BD, Beyene AS, Bhala N, Bhansali A, Bhatt S, Biadgilign S, Bikbov B, Bisanzio D, Bjertness E, Blore JD, Borschmann R, Boufous S, Bourne RRA, Brainin M, Brazinova A, Breitborde NJK, Brenner H, Broday DM, Brugha TS, Brunekreef B, Butt ZA, Cahill LE, Calabria B, Campos-Nonato IR, Cárdenas R, Carpenter DO, Carrero JJ, Casey DC, Castañeda-Orjuela CA, Rivas JC, Castro RE, Catalá-López F, Chang JC, Chiang PPC, Chibalabala M, Chimed-Ochir O, Chisumpa VH, Chitheer AA, Choi JYJ, Christensen H, Christopher DJ, Ciobanu LG, Coates MM, Colquhoun SM, Manzano AGC, Cooper LT, Cooperrider K, Cornaby L, Cortinovis M, Crump JA, Cuevas-Nasu L, Damasceno A, Dandona R, Darby SC, Dargan PI, das Neves J, Davis AC, Davletov K, de Castro EF, de la Cruz-Góngora V, de Leo D, Degenhardt L, del Gobbo LC, del Pozo-Cruz B, Dellavalle RP, Deribew A, Jarlais DCD, Dharmaratne SD, Dhillon PK, Diaz-Torné C, Dicker D, Ding EL, Dorsey ER, Doyle KE, Driscoll TR, Duan L, Dubey M, Duncan BB, Elyazar I, Endries AY, Ermakov SP, Erskine HE, Eshrati B, Esteghamati A, Fahimi S, Faraon EJA, Farid TA, Farinha CSS, Faro A, Farvid MS, Farzadfar F, Feigin VL, Fereshtehnejad SM, Fernandes JG, Fischer F, Fitchett JRA, Fleming T, Foigt N, Foreman K, Fowkes FGR, Franklin RC, Fürst T, Futran ND, Gakidou E, Garcia-Basteiro AL, Gebrehiwot TT, Gebremedhin AT, Geleijnse JM, Gessner BD, Giref AZ, Giroud M, Gishu MD, Giussani G, Goenka S, Gomez-Cabrera MC, Gomez-Dantes H, Gona P, Goodridge A, Gopalani SV, Gotay CC, Goto A, Gouda HN, Gugnani HC, Guillemin F, Guo Y, Gupta R, Gupta R, Gutiérrez RA, Haagsma JA, Hafezi-Nejad N, Haile D, Hailu GB, Halasa YA, Hamadeh RR, Hamidi S, Handal AJ, Hankey GJ, Hao Y, Harb HL, Harikrishnan S, Haro JM, Hassanvand MS, Hassen TA, Havmoeller R, Heredia-Pi IB, Hernández-Llanes NF, Heydarpour P, Hoek HW, Hoffman HJ, Horino M, Horita N, Hosgood HD, Hoy DG, Hsairi M, Htet AS, Hu G, Huang JJ, Husseini A, Hutchings SJ, Huybrechts I, Iburg KM, Idrisov BT, Ileanu BV, Inoue M, Jacobs TA, Jacobsen KH, Jahanmehr N, Jakovljevic MB, Jansen HAFM, Jassal SK, Javanbakht M, Jayaraman SP, Jayatilleke AU, Jee SH, Jeemon P, Jha V, Jiang Y, Jibat T, Jin Y, Johnson CO, Jonas JB, Kabir Z, Kalkonde Y, Kamal R, Kan H, Karch A, Karema CK, Karimkhani C, Kasaeian A, Kaul A, Kawakami N, Kazi DS, Keiyoro PN, Kemmer L, Kemp AH, Kengne AP, Keren A, Kesavachandran CN, Khader YS, Khan AR, Khan EA, Khan G, Khang YH, Khatibzadeh S, Khera S, Khoja TAM, Khubchandani J, Kieling C, Kim CI, Kim D, Kimokoti RW, Kissoon N, Kivipelto M, Knibbs LD, Kokubo Y, Kopec JA, Koul PA, Koyanagi A, Kravchenko M, Kromhout H, Krueger H, Ku T, Defo BK, Kuchenbecker RS, Bicer BK, Kuipers EJ, Kumar GA, Kwan GF, Lal DK, Lalloo R, Lallukka T, Lan Q, Larsson A, Latif AA, Lawrynowicz AEB, Leasher JL, Leigh J, Leung J, Levi M, Li X, Li Y, Liang J, Liu S, Lloyd BK, Logroscino G, Lotufo PA, Lunevicius R, MacIntyre M, Mahdavi M, Majdan M, Majeed A, Malekzadeh R, Malta DC, Manamo WAA, Mapoma CC, Marcenes W, Martin RV, Martinez-Raga J, Masiye F, Matsushita K, Matzopoulos R, Mayosi BM, McGrath JJ, McKee M, Meaney PA, Medina C, Mehari A, Mejia-Rodriguez F, Mekonnen AB, Melaku YA, Memish ZA, Mendoza W, Mensink GBM, Meretoja A, Meretoja TJ, Mesfin YM, Mhimbira FA, Millear A, Miller TR, Mills EJ, Mirarefin M, Misganaw A, Mock CN, Mohammadi A, Mohammed S, Mola GLD, Monasta L, Hernandez JCM, Montico M, Morawska L, Mori R, Mozaffarian D, Mueller UO, Mullany E, Mumford JE, Murthy GVS, Nachega JB, Naheed A, Nangia V, Nassiri N, Newton JN, Ng M, Nguyen QL, Nisar MI, Pete PMN, Norheim OF, Norman RE, Norrving B, Nyakarahuka L, Obermeyer CM, Ogbo FA, Oh IH, Oladimeji O, Olivares PR, Olsen H, Olusanya BO, Olusanya JO, Opio JN, Oren E, Orozco R, Ortiz A, Ota E, PA M, Pana A, Park EK, Parry CD, Parsaeian M, Patel T, Caicedo AJP, Patil ST, Patten SB, Patton GC, Pearce N, Pereira DM, Perico N, Pesudovs K, Petzold M, Phillips MR, Piel FB, Pillay JD, Plass D, Polinder S, Pond CD, Pope CA, Pope D, Popova S, Poulton RG, Pourmalek F, Prasad NM, Qorbani M, Rabiee RHS, Radfar A, Rafay A, Rahimi-Movaghar V, Rahman M, Rahman MHU, Rahman SU, Rai RK, Rajsic S, Raju M, Ram U, Rana SM, Ranganathan K, Rao P, García CAR, Refaat AH, Rehm CD, Rehm J, Reinig N, Remuzzi G, Resnikoff S, Ribeiro AL, Rivera JA, Roba HS, Rodriguez A, Rodriguez-Ramirez S, Rojas-Rueda D, Roman Y, Ronfani L, Roshandel G, Rothenbacher D, Roy A, Saleh MM, Sanabria JR, Sanchez-Riera L, Sanchez-Niño MD, Sánchez-Pimienta TG, Sandar L, Santomauro DF, Santos IS, Sarmiento-Suarez R, Sartorius B, Satpathy M, Savic M, Sawhney M, Schmidhuber J, Schmidt MI, Schneider IJC, Schöttker B, Schutte AE, Schwebel DC, Scott JG, Seedat S, Sepanlou SG, Servan-Mori EE, Shaddick G, Shaheen A, Shahraz S, Shaikh MA, Levy TS, Sharma R, She J, Sheikhbahaei S, Shen J, Sheth KN, Shi P, Shibuya K, Shigematsu M, Shin MJ, Shiri R, Shishani K, Shiue I, Shrime MG, Sigfusdottir ID, Silva DAS, Silveira DGA, Silverberg JI, Simard EP, Sindi S, Singh A, Singh JA, Singh PK, Slepak EL, Soljak M, Soneji S, Sorensen RJD, Sposato LA, Sreeramareddy CT, Stathopoulou V, Steckling N, Steel N, Stein DJ, Stein MB, Stöckl H, Stranges S, Stroumpoulis K, Sunguya BF, Swaminathan S, Sykes BL, Szoeke CEI, Tabarés-Seisdedos R, Takahashi K, Talongwa RT, Tandon N, Tanne D, Tavakkoli M, Taye BW, Taylor HR, Tedla BA, Tefera WM, Tegegne TK, Tekle DY, Terkawi AS, Thakur JS, Thomas BA, Thomas ML, Thomson AJ, Thorne-Lyman AL, Thrift AG, Thurston GD, Tillmann T, Tobe-Gai R, Tobollik M, Topor-Madry R, Topouzis F, Towbin JA, Tran BX, Dimbuene ZT, Tsilimparis N, Tura AK, Tuzcu EM, Tyrovolas S, Ukwaja KN, Undurraga EA, Uneke CJ, Uthman OA, van Donkelaar A, van Os J, Varakin YY, Vasankari T, Veerman JL, Venketasubramanian N, Violante FS, Vollset SE, Wagner GR, Waller SG, Wang JL, Wang L, Wang Y, Weichenthal S, Weiderpass E, Weintraub RG, Werdecker A, Westerman R, Whiteford HA, Wijeratne T, Wiysonge CS, Wolfe CDA, Won S, Woolf AD, Wubshet M, Xavier D, Xu G, Yadav AK, Yakob B, Yalew AZ, Yano Y, Yaseri M, Ye P, Yip P, Yonemoto N, Yoon SJ, Younis MZ, Yu C, Zaidi Z, Zaki MES, Zhu J, Zipkin B, Zodpey S, Zuhlke LJ, Murray CJL (2016). Global, regional, and national comparative risk assessment of 79 behavioural, environmental and occupational, and metabolic risks or clusters of risks, 1990-2015: a systematic analysis for the global burden of disease study 2015. Lancet.

[5] Stea TH, Nordheim O, Bere E, Stornes P, Eikemo TA (2020). Fruit and vegetable consumption in Europe according to gender, educational attainment and regional affiliation-A cross-sectional study in 21 European countries. PLoS One.

[6] Prättälä R, Paalanen L, Grinberga D, Helasoja V, Kasmel A, Petkeviciene J (2006). Gender differences in the consumption of meat, fruit and vegetables are similar in Finland and the Baltic countries. Eur J Pub Health.

[7] Mensink GBM, Schienkiewitz A, Lange C. Gemüsekonsum bei Erwachsenen in Deutschland. Journal of Health Monitoring. 2017;2(2).

[8] WHO Fruit and Vegetable Promotion Initiative – report of the meeting. Geneva: World Health Organization; 2003.

[9] Johnson J, Repta R. Sex and Gender: Beyond the Binaries. In: Oliffe JL GL, editor. Designing and conducting gender, sex, and health research Los Angeles/London/New Dehli/Singapore/ Washington DC: SAGE Publications; 2012. p. 17–37.

[10] Bauer GR (2014). Incorporating intersectionality theory into population health research methodology: challenges and the potential to advance health equity. Soc Sci Med.

[11] Breiman L, Friedman J, Olshen R, Stone C (1984). Classification and regression trees.

[12] Bauer GR, Churchill SM, Mahendran M, Walwyn C, Lizotte D, Villa-Rueda AA (2021). Intersectionality in quantitative research: a systematic review of its emergence and applications of theory and methods. SSM Popul Health.

[13] Mubayi A. Chapter 10 - Computational Modeling Approaches Linking Health and Social Sciences: Sensitivity of Social Determinants on the Patterns of Health Risk Behaviors and Diseases. In: Srinivasa Rao ASR, Pyne S, Rao CR, editors. Handbook of Statistics. 36: Elsevier; 2017. p. 249–304.

[14] Hothorn T, Hornik K, Zeileis A (2006). Unbiased recursive partitioning: a conditional inference framework. J Comput Graph Stat.

[15] Henrard S, Speybroeck N, Hermans C (2015). Classification and regression tree analysis vs. multivariable linear and logistic regression methods as statistical tools for studying haemophilia. Haemophilia..

[16] Lemon SC, Roy J, Clark MA, Friedmann PD, Rakowski W (2003). Classification and regression tree analysis in public health: methodological review and comparison with logistic regression. Ann Behav Med.

[17] Friel SN (2004). John; Kelleher, Cecily. Who eats four or more servings of fruit and vegetables per day? Multivariate classification tree analysis of data from the 1998 survey of lifestyle, attitudes and nutrition in the Republic of Ireland. Public Health Nutr.

[18] Kim S, Choi M-K (2014). Factors associated with fruit and vegetable consumption of subjects having a history of stroke: using 5th Korea National Health and nutrition examination survey (2010, 2011). Korean J Community Nutr.

[19] RKI. Gesundheit in Deutschland Aktuell 2009. public use file. GEDA 2009 Dokumentation des Datensatzes 2011.

[20] RKI. Daten und Fakten: Ergebnisse der Studie „Gesundheit in Deutschland aktuell 2010″. Berlin: RKI; 2012.

[21] Schenk L, Ellert U, Neuhauser H (2007). Kinder und Jugendliche mit Migrationshintergrund in Deutschland. Bundesgesundheitsblatt Gesundheitsforschung Gesundheitsschutz.

[22] Johnson JL, Greaves, L., & Repta, R. Better Science with Sex and Gender: A Primer for Health Research2007. 10.1186/1475-9276-8-14 PMC2689237 19419579

[23] Pelletier R, Ditto B, Pilote L (2015). A composite measure of gender and its association with risk factors in patients with premature acute coronary syndrome. Psychosom Med.

[24] Mena E, Bolte G (2019). on behalf of the ADVANCE GENDER Study Group. Intersectionality-based quantitative health research and sex/gender sensitivity: a scoping review. Int. J. Equity Health.

[25] Mena E (2021). Bolte G, on behalf of the ADVANCE GENDER study group. CART-analysis embedded in social theory: A case study comparing quantitative data analysis strategies for intersectionality-based public health monitoring within and beyond the binaries SSM Popul Health.

[26] Dalgard OS, Bjørk S, Tambs K (1995). Social support, negative life events and mental health. Br J Psychiatry.

[27] Meltzer H (2003). Development of a common instrument for mental health. In: A. Nosikov, Gudex C, editors. EUROHIS: developing common instruments for health surveys.

[28] Therneau TM (2019). Atkinson.

[29] Hothorn T, Zeileis A (2015). Partykit: a modular toolkit for recursive Partytioning in R. J Mach Learn Res.

[30] R Core Team. A language and environment for statistical computing. Vienna, Austria 2013. http://www.R-project.org/.

[31] Venkatasubramaniam A, Wolfson J, Mitchell N, Barnes T, JaKa M, French S (2017). Decision trees in epidemiological research. Emerg Themes Epidemiol.

[32] Cairney J, Veldhuizen, S., Vigod, S., Streiner, D.L., Wade, T.J., & Kurdyak, P. Exploring the social determinants of mental health service use using intersectionality theory and CART analysis. J Epidemiol Community Health 2014;68:145–150, 2, DOI: 10.1136/jech-2013-203120. 10.1136/jech-2013-203120 24098046

[33] Emanuel AS, McCully SN, Gallagher KM, Updegraff JA (2012). Theory of planned behavior explains gender difference in fruit and vegetable consumption. Appetite..

[34] Turrell G, Hewitt B, Patterson C, Oldenburg B (2003). Measuring socio-economic position in dietary research: is choice of socio-economic indicator important?. Public Health Nutr.

[35] Galobardes B, Morabia A, Bernstein MS (2001). Diet and socioeconomic position: does the use of different indicators matter?. Int J Epidemiol.

[36] Barrington WE, Ceballos RM, Bishop SK, McGregor BA, Beresford SAA (2012). Perceived stress, behavior, and body mass index among adults participating in a worksite obesity prevention program, Seattle, 2005–2007. Prev Chronic Dis.

[37] Gardiner CK, Hagerty SL, Bryan AD. Stress and number of servings of fruit and vegetables consumed: buffering effects of monetary incentives. J Health Psychol 2019;0(0):1359105319884620, 26, 10, 1359105319881763, DOI: 10.1177/1359105319884620. 10.1177/1359105319884620 31665933

[38] Dėdelė A, Miškinytė A, Andrušaitytė S, Bartkutė Ž (2019). Perceived stress among different occupational groups and the interaction with sedentary behaviour. Int J Environ Res Public Health.

[39] Guinea-Martin D, Mora R, Ruiz-Castillo J (2018). The evolution of gender segregation over the life course. Am Sociol Rev.

[40] Campos-Serna J, Ronda-Pérez E, Artazcoz L, Moen BE, Benavides FG (2013). Gender inequalities in occupational health related to the unequal distribution of working and employment conditions: a systematic review. Int J Equity Health.

[41] Hammarström A, Johansson K, Annandale E, Ahlgren C, Alex L, Christianson M (2014). Central gender theoretical concepts in health research: the state of the art. J Epidemiol Community Health.

[42] Elser H, Falconi AM, Bass M, Cullen MR (2018). Blue-collar work and women's health: a systematic review of the evidence from 1990 to 2015. SSM Popul Health..

[43] Arias-de la Torre J, Molina AJ, Fernández-Villa T, Artazcoz L, Martín V (2019). Mental health, family roles and employment status inside and outside the household in Spain. Gac Sanit.

[44] Al AD, Anıl İ (2016). The comparison of the individual performance levels between full-time and part-time employees: the role of job satisfaction. Procedia Soc Behav Sci.

[45] Cheung KL, ten Klooster PM, Smit C, de Vries H, Pieterse ME (2017). The impact of non-response bias due to sampling in public health studies: a comparison of voluntary versus mandatory recruitment in a Dutch national survey on adolescent health. BMC Public Health.

[46] Magallares A (2016). Drive for thinness and pursuit of muscularity: the role of gender ideologies. Univ Psychol.

[47] Pivonka E, Seymour J, McKenna J, Baxter SD, Williams S (2011). Development of the Behaviorally Focused Fruits & Veggies—More Matters Public Health Initiative. J Am Diet Assoc.

[48] Pöge K, Rommel A, Mena E, Holmberg C, Saß A-C, Bolte G (2019). AdvanceGender – Verbundprojekt für eine geschlechtersensible und intersektionale Forschung und Gesundheitsberichterstattung. Bundesgesundheitsblatt Gesundheitsforschung Gesundheitsschutz..

[49] Lange C, Jentsch F, Allen J, Hoebel J, Kratz AL, von der Lippe E, Muters S, Schmich P, Thelen J, Wetzstein M, Fuchs J, Ziese T (2015). Data resource profile: German health update (GEDA)—the health interview survey for adults in Germany. Int J Epidemiol.

