# Supplementary material for: Classification tree analysis for an intersectionality-informed identification of population groups with non-daily vegetable intake
Source: BMC Public Health. 2021 Nov 4;21:2007. doi: 10.1186/s12889-021-12043-6 (PMC8570019; doi:10.1186/s12889-021-12043-6)
Supplement: Supplementary file 2 — Additional file 2. [file 12889_2021_12043_MOESM2_ESM.docx]

**Additional file 2**


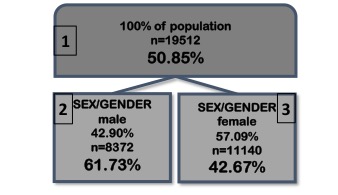


Strategy 1 - Splitting variables, proportion of study population and prevalence of non-DVI within subgroups detected by CART-analysis (cp = 0.01; cp = 0.005) based on binary sex/gender variable and socio-cultural, socio-demographic and socio-economic variables of the full sample.
